# Infantile myofibromatosis and capillary malformation of the skin due to *PDGFRB* mosaicism

**DOI:** 10.1186/s40348-025-00197-x

**Published:** 2025-07-09

**Authors:** Luise Pudig, Silke Lassmann, Sebastian Jacob, Marina Nastainczyk-Wulf, Anja Haak, Martin Werner, Friedrich G Kapp, Simone Hettmer

**Affiliations:** 1 University Medicine Halle, Martin Luther University Halle, Pediatrics 1, Halle (Saale), Germany; 2 Institute for Surgical Pathology, Medical Center, University of Freiburg, Freiburg, Germany; 3 Comprehensive Cancer Center Freiburg, Medical Center, Freiburg, Germany; 4 Center for Personalized Medicine, Partner Site Freiburg, Freiburg, Germany; 5 Institute of Legal Medicine, University Medicine Halle, Halle (Saale), Germany; 6 Institute of Pathology, University Medicine Halle, Halle (Saale), Germany; 7 German Cancer Consortium (DKTK), partner site Freiburg, Freiburg, Germany; 8 Department of Pediatric Hematology and Oncology, Medical Center, Faculty of Medicine, Children’s Hospital, University of Freiburg, Freiburg, Germany; 9 Krukenberg Cancer Center, Martin Luther University Halle, Halle (Saale), Germany

## Abstract

This report describes the case of a 25-year-old female patient with multicentric infantile myofibromatosis since early infancy, superficial capillary malformations and congenital hypoplasia of the third and fourth finger of her right hand. All known lesions were located in the upper extremities, the chest and the upper back. A pathogenic, gain-of-function platelet-derived growth factor receptor-beta (*PDGFRB)* variant (p.N666K, c.1998 C > A) was detected in two myofibromas and in a capillary malformation on the upper back, but not in DNA obtained from blood mononuclear cells. Thus, *PDGFRB* mosaicism appears to account for the patient’s myofibromas and capillary malformations, supporting a broad spectrum of *PDGFRB*-driven anomalies ranging from myofibromas to vascular malformations.

## Introduction

Infantile myofibromatosis (IM) is a rare condition, accounting for the majority of soft tissue tumors diagnosed during infancy [1]. IM is characterized by nodules of the skin, subcutaneous tissues, muscles and less often bones or visceral organs. IM typically regresses spontaneously, but visceral involvement in up to 19% of all cases may cause life-threatening complications [2]. IM has been associated with somatic, mosaic or germline gain-of-function variants in the platelet-derived growth factor receptor-beta (*PDGFRB*) gene [3]. Germline *PDGFRB* variants may cause Penttinen or Kosaki overgrowth syndrome, but variants associated with these conditions overactivate PDGFRB more potently than those linked to familial IM [4]. *PDGFRB* variants were also associated with aneurysm formation [5–9]and several patients with IM and (intracerebral) aneurysms were documented in the literature [7]. Here, we report on a 25-year-old woman with IM and capillary malformations (CMs) associated with *PDGRFB* p.Asn666Lys mosaicism. This case provides evidence that aberrant PDGFB signaling causes a broad phenotypic spectrum of anomalies ranging from myofibromas to vascular malformations.

## Case report

**Fig. 1:**
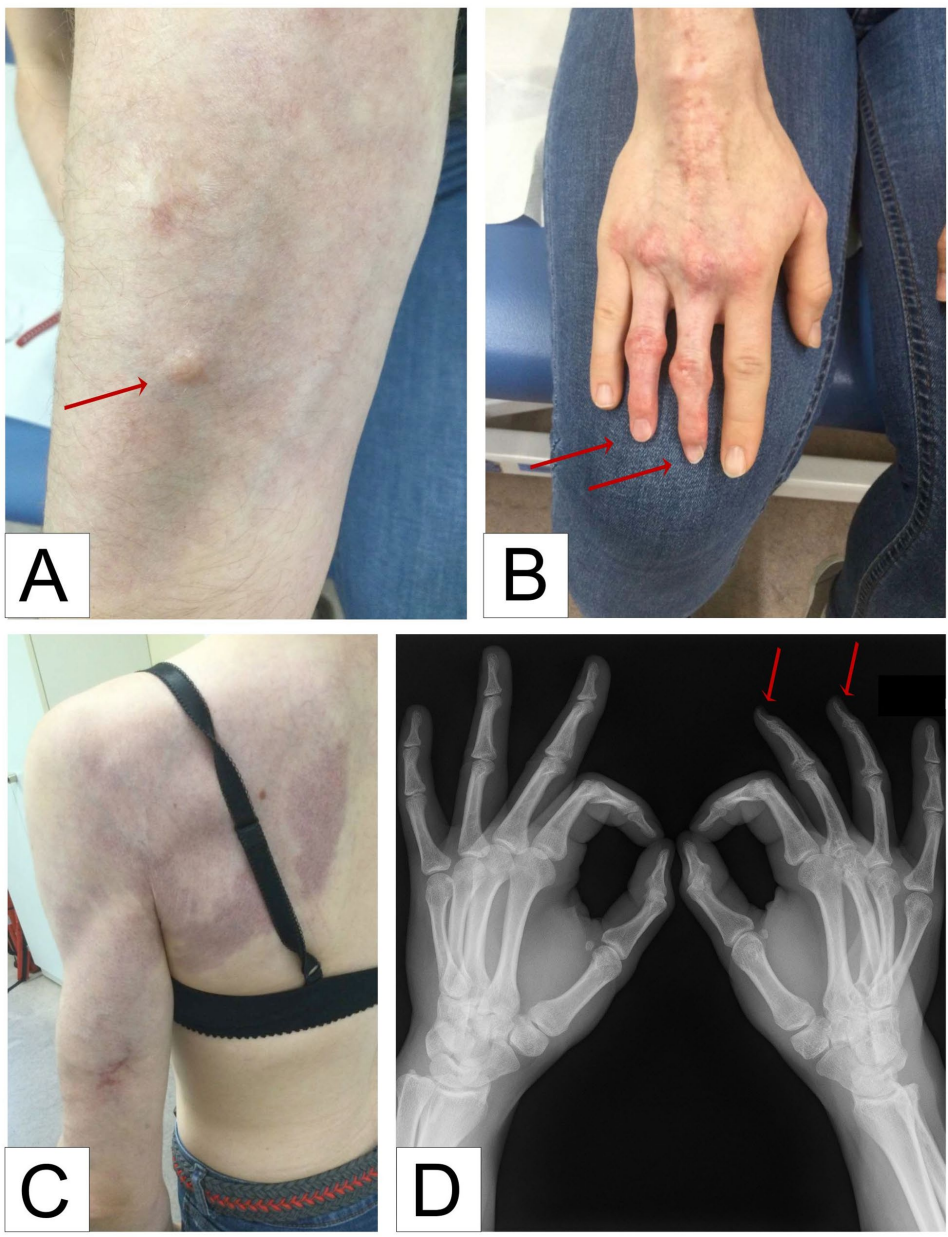
25-year-old young woman with multiple myofibromas and vascular anomalies, including **(A)** a superficial myofibroma of the right forearm, **(B)** congenital hypoplasia of the third and fourth finger of the right hand and **(C)** capillary malformation of the skin. **(D)** Xrays of both hands show distal mutilation of the metatarsal bones and the phalanges of the third and forth digit of the right hand

A 25-year-old female patient was diagnosed with infantile myofibromatosis (IM) in early infancy. Resection of a nodule on her upper back and biopsy of another nodule on the back of her right hand confirmed IM histology. In the following years, new superficial nodules developed, others increased in size or disappeared spontaneously (Fig. 1A). Clinical signs of visceral IM involvement or ipsilateral overgrowth were never noted. Cutaneous manifestations including livid discoloration and increased vascular patterning on the skin of her arms, hands, chest and upper back were noted early on (Fig. 1B). Finally, congenital hypoplasia of the third and fourth finger of her right hand has been associated with radiological changes indicative of malperfusion (Fig. 1C) and complicated by secondary inflammation and prolonged wound healing throughout her life.

DNA sequencing was performed by large Panel-NGS (TruSight Oncology 500, Illumina) according to accredited molecular pathology diagnostics using DNA obtained from formalin-fixed and paraffin-embedded tissue samples obtained in 2008 (myofibroma), 2011 (myofibroma) and 2023 (CM of the skin). In addition to several synonymous, intronic, benign missense or 3’-UTR variants, the likely pathogenic PDGFRB variant p.N666K (c.1998 C > A) – which was previously linked to IM [4] - was detected in all three specimen (Table 1, bold font). Variant allele frequencies were 46% (2008 lesion), 26% (2011 lesion) and 18% (2023 lesion). By contrast, next-generation sequencing of DNA isolated from mononuclear blood cells and Sanger-sequencing of DNA obtained from fingernail sections from a hypoplastic finger of the patient did not reveal the previously identified *PDGFRB* variant. There was no family history of IM, but the patient’s paternal uncle died suddenly at 30 years of age.

**Table 1:** PDGFRB variants detected in a myofibroma resected in 2008 (IM #1), a myofibroma resected in 2011 (IM #2) and a capillary malformation of the skin (CM) biopsied in 2023. All detected PDGFRB variants are listed and specified according to the human genome variation society (HGVS) nomenclature. Variant dignitiy was estimated using the databases clinvar [23] and intervar [24] as well as the REVEL [25] algorithm and ACGME criteria [26, 27]. Abbreviations: IM - Infantile myofibromatosis; HGVSc - human genome variation society coding; Cov – coverage; VAF - variant allele frequency; 3’UTR - three prime untranslated region; P/LP – pathogenic /likely pathogenic; LB – likely benign

Specimen	Variant (HGVSc)	Cov	VAF (%)	Consequence	Clin Var	Inter Var	REVEL	ACMG
IM #1	NM_002609.3:c.*1854G > A	55	100	3’UTR				1
	NM_002609.3:c.3252 A > G	133	41,35	synonymous				1
	NM 002609.3:c.3090 C > T	111	52,25	synonymous				1
	NM_002609.3:c2253T > C	122	4,1	synonymous				1
	**NM_002609.3:c.1998 C > A**	**105**	**40**,**95**	**missense**	**P/LP**	**LP**	**0**,**67**	**5**
	NM_002609.3:c.1453G > A	130	46,15	missense	LB			1
	NM_002609.3:c-6–2208 A > C	233	95,71	intron				1
	NM 002609.3:c.-6-7161G > A	252	99,6	intron				1
IM #2	NM_002609.4:c.*1854G > A	11	100	3’UTR				1
	NM_002609.4:c.3252 A > G	60	58,33	synonymous				1
	NM_002609.4:c.3090 C > T	58	43,1	synonymous				1
	**NM_002609.4:c.1998 C > A**	**51**	**25**,**49**	**missense**	**P/LP**	**LP**	**0**,**67**	**5**
	NM_002609.4:c.1453G > A	64	32,81	missense	LB			1
	NM_002609.4:c.-6–2208 A > C	79	100	intron				1
	NM_002609.4:c.-6-7161G > A	85	100	intron				1
CM	NM_002609.4:c.*1854G > A	194	100	3’UTR				1
	NM_002609.4:c.3252 A > G	344	49,42	synonymous				1
	NM_002609.4:c.3090 C > T	314	53,82	synonymous				1
	**NM_002609.4:c.1998 C > A**	**308**	**17**,**86**	**missense**	**P/LP**	**LP**	**0**,**67**	**5**
	NM_002609.4:c.1453G > A	623	47,67	missense	LB			1
	NM_002609.4:c.-6–2208 A > C	332	100	intron				1
	NM_002609.4:c.-6-7161G > A	377	100	intron				1

At the age of 25, the patient continues to have several myofibromas and diffuse CMs, which are located on her arms, chest and upper back. Myofibromas or cutaneous manifestations were never noted in any other body parts. X-ray of the fingers showed osseous remodelling of the third and fourth finger of the right hand with atypical bone structure and distal mutilation of the metatarsal bones and the phalanges (Fig. 1d). Whole body magnetic resonance imaging (MRI) and dedicated MRI of the brain did not identify aneurysms or evidence of visceral IM. The patient is a lifeguard and enrolled in a Master’s program at a German university; quality of life is excellent. She provided written informed consent for the report of her case.

## Discussion

Here, we report on a 25-year-old young woman with IM, CMs of the skin and hypoplasia of two fingers of the right hand associated with *PDGFRB* p.N666K mosaicism. The case highlights co-occurrence of IM and CMs due to a mosaic gain-of-function *PDGFRB* variant, which was previously linked to IM [4]. Hypoplasia of two fingers of the right hand has been associated with radiographic changes indicative of malperfusion. Alternatively, it is possible that PDGFRB mosaicism directly contributed to the finger anomalies, as skeletal abnormalities including acroosteolysis have been described in individuals with Penttinen or Kosaki syndrome (typically due to germline gain-of-function PDGFRB variants resulting in amino acid 665 changes) [10]. 

Strikingly, all disease manifestations in this patient are localized in the upper extremities, chest and upper back, which is consistent with type 1 segmental mosaicism due to a postzygotic mutation in somatic cells during early embryonic development. It is typically associated with rather large, segmental lesions [11]. Type 1 mosaic activating mutations of mTOR/PIK3CA- or RAS-MAPK-pathway genes are thought to underlie most vascular malformation syndromes [12]. 

PDGFRB is a receptor tyrosine kinase (RTK), which mediates its proliferative effects mainly via the RAS/RAF/Erk- and PIK/Akt/mTOR-pathways [13]. Recent studies suggest a central role of somatic *PDGFRB* mutations in the development of infantile hemangiomas [14–16] and (intracerebral) aneurysms [5, 6, 8]. This causative association is plausible, since aberrant RAS/RAF/Erk- and PI3K/Akt/mTOR-signalling has been well characterized as the driving pathomechanism behind the formation of syndromic vascular malformations as well as malign transformation [17, 18]. To our best knowledge, *PDGFRB* variants have not been linked to CMs before.

In the recent literature, CM have been associated with aberrant PI3K signal transduction [19, 20] and aberrant GPCR signal transduction due to pathogenic *GNAQ* or *GNA11* mutations [18, 21, 22]. This is in line with the pleiotropic functions of *GNAQ* and *GNA11* in cell signalling, mediating crosstalk between GPCR and RTK cascades. The full spectrum of genetic variants contributing to the development of CMs and other vascular anomalies remains to be discovered.

The PDGFRB inhibitor imatinib, has shown therapeutic promise in managing severe phenotypes of *PDGFRB*-driven disorders, ranging from IM to Penttinen or Kosaki syndrome [10]. In fact, myofibromas linked to the *PDGFRB* p.N666K variant – the specific mutation detected in our patient’s myofibromas and CM – was shown to respond to imatinib. As quality of life is excellent and the patient may consider family planning soon, imatinib treatment was dismissed in our patient. In case of functional or cosmetic impairment due to myofibroma growth or malperfusion, treatment with imatinib could be considered in the future.

Our observations in the patient reported here suggest that aberrant PDGFRB signaling causes a wide phenotypic spectrum ranging from myofibromas to vascular malformations and support the notion that individuals suffering from IM due to germline or mosaic *PDGFRB* variants are at risk of developing vascular complications.

## Data Availability

No datasets were generated or analysed during the current study.

## Abbreviations

CM: Capillary malformation
IM: Infantile myofibromatosis
PDGRFB: Platelet-derived growth factor receptor-beta
MRI: Magnetic resonance imaging
PWS: Port-wine stains
RTK: Receptor tyrosine kinase
